# Transparent peer review trial: the results

**DOI:** 10.1186/s13059-018-1584-0

**Published:** 2018-11-27

**Authors:** Andrew Cosgrove, Barbara Cheifet

**Affiliations:** Genome Biology, London, UK

## Abstract

We describe the results of our year-long trial of transparent peer review and announce the adoption of transparent review as permanent policy.

A year ago, *Genome Biology* published an editorial describing a trial of transparent review at the journal (where transparent review is the process of publishing reviewer reports anonymously alongside the published article) [1]. Other journals already use this model of peer review and have reported reasonably high author satisfaction [2], but we could find no quantitative reports about the effects on manuscript turnaround times or the engagement of peer reviewers. We were concerned that reviewers might be more reluctant to agree to review if they knew that their reviews would be published. This would lead to increased turnaround times for authors, since a large factor in determining the time elapsed between submission and the initial decision is the length of time taken to find sufficient numbers of reviewers to agree to review. Thus, we started a trial to compare submissions assessed under traditional single-blind review (where the authors’ identities are known to reviewers, the reviewers remain anonymous, and their reports are not published) with those reviewed transparently.

We have now assessed the data we collected over the year. In the trial, 45 submissions underwent transparent review for at least one round of review, and 68 went through traditional single-blind peer review. Eligible authors could opt out of the trial before the review procedure commenced either explicitly or passively (if they did not reply to our emails), which accounts for the difference in numbers between the two arms. Our main finding is that there was no significant difference in the mean time to first decision between transparent review and single-blind review. For both sides of the trial, the average number of reviewers we needed to invite for each agreed reviewer was 3.1. Thus, we can see no evidence that reviewers are more reluctant to agree to review transparently. This is also borne out in the responses we received from reviewers—those who commented at all were positive; no reviewer declined to review explicitly because of transparency, although we cannot rule out that some declined for this reason without letting us know.

A low rate of reviewer engagement is only one potential downside of transparent review, though. Another concern might be that reviewers would be reluctant to be critical knowing that their criticisms would be published; however, we see no evidence of that in our data. There is no significant difference between the two arms of the trial in the proportions of manuscripts given a first decision of outright rejection, rejection with the option of resubmitting a revised version, or major revisions. Of those manuscripts that have been through the trial to a final decision (published or rejected), there is no difference in the overall rejection rate.

Based on these results, it seems to us that transparent review does not affect the speed or outcome of the peer-review process. It should be noted, however, that the pool of transparently reviewed manuscripts had some element of self-selection, as authors whose manuscripts qualified to be transparently reviewed were offered the option to take part (or not) in the trial. This might introduce some bias to the results. For example, authors who suspect that their studies might be reviewed more negatively might opt for traditional review, as they might not want criticisms of their paper to be made public. Whether or not this bias affected the results, it is true that 13 out of 98 authors chose not to take part in the trial. Somewhat to our surprise, three of those 13 explicitly stated that they opted out because review would not be fully open (suggesting that they would wish the reviewers’ names to be published alongside the reviews). Moving to transparent review permanently would therefore run the risk of putting off some authors and reducing the number of manuscripts submitted to the journal.

One criticism of traditional peer review is that a reader does not know who the reviewers were, and so cannot judge whether they had sufficient expertise to assess the manuscript, thus meaning the stamp of ‘peer reviewed’ is of uncertain value. Although transparent review does not fully address this, since the reader still will not know the reviewers’ identities, the reader will be able to read the comments and assess whether the reviewer has made sensible and reasonable criticism of the work, which should lead to increased confidence in the peer-review assessment. Fully open review would be even better from this point of view, but many reviewers may be reluctant to associate their names with negative reviews, even when the negative comments are justified, for fear of retaliation. It seems to us that transparent review is currently the best compromise, and we hope that, when it is well established, it will foster a more open environment where reviewers will feel more comfortable in revealing their identities.

Therefore, we feel that transparent review will be the right choice for the journal. Our results suggest some authors might be reluctant to submit under those circumstances, but we nevertheless feel that the benefits of transparent review outweigh a possible small drop in submissions. We will be making this move permanently: after 1 January 2019, all submissions will be reviewed transparently. By doing this, we join an increasing number of journals that are adopting transparency. Recently, editors from over 100 journals from a variety of publishers signed an open letter committing to move to transparent review [3]. We are excited about joining them.
